# Echoes of Telecardiology Guideline

**DOI:** 10.36660/abc.20190720

**Published:** 2020-01

**Authors:** Silvio Henrique Barberato, Marcelo Antônio Cartaxo Queiroga Lopes

**Affiliations:** 1 CardioEco Centro de Diagnóstico Cardiovascular, Curitiba, PR - Brazil; 2 Quanta Diagnóstico - Ecocardiografia, Curitiba, PR - Brazil; 3 Sociedade Brasileira de Cardiologia - Departamento de Imagem Cardiovascular, São Paulo, SP - Brazil; 4 Sociedade Brasileira de Cardiologia, Rio de Janeiro, RJ - Brazil; 5 Hospital Alberto Urquiza Wanderley, João Pessoa, PB - Brazil

**Keywords:** Cardiovascular Diseases, Diagnosis imaging, Echocardiography/methods, Telemedicine/methods, Telemedicine/trends, Robotics/trends, Training, Image Interpretation Computer-Assisted, Telemonitoring

Echocardiography has an established role in the diagnosis, prognostic evaluation and therapeutic orientation in several cardiovascular diseases.^1^ The great technological development in the last decades has allowed the digitization and standardization of medical images (DICOM), miniaturization of echocardiography equipment (portable cardiac ultrasound) and the transfer of images over the internet. In this scenario, strategies for the use of telemedicine-associated echocardiography, called tele-echocardiography, have been employed in the context of clinical research with the support of teleconsultation for remote expert review, in real time or after image storage and submission. In recognition of the demands generated by the ongoing digital transformation in health, the Brazilian Society of Cardiology recently published the Telemedicine Guideline in Cardiology.^2^ The document recognizes tele-echocardiography as a strategy for early detection of congenital heart disease in newborns and screening for early detection of subclinical cases of rheumatic heart disease in children and adolescents (both recommended as indication class IIa, level of evidence B). In addition, it evokes potential application in primary health care in remote locations, where it could enable early detection of cases of heart disease and assist in prioritizing referrals to specialized care (indication class IIb, level of evidence C).^2^ It should be emphasized that such recommendations are made within the conditions of regular use of the method in Brazil, which would include the need for doctors at both ends, i.e., the execution and interpretation of the exam. The text of the Guideline explicitly states the need for regulation and legal provision for the participation of other professionals in performing diagnostic procedures (in this case, obtaining echocardiographic images by non-doctors), currently not allowed by the country’s legislation.

In recent years, tele-echocardiography has extended the application of the method to individuals in geographically distant locations, such as remote rural communities^3^ or even space.^4^ A rural area is classified as remote when 50% of the local population needs at least 45 to 60 minutes of travel by motor vehicle to reach a population center of at least 50,000 inhabitants.^2^ Several situations have been experimentally tested, with a study on focused echocardiographic by non-cardiologist physicians,^5,6^ non-physicians,^3,7^ or remote-operated robotic devices,^8^ combined with remote interpretation by echocardiography cardiologists.

Historically, tele-echocardiography was initially employed in pediatric populations to rule out relevant congenital heart disease, with either live or offline transmission approaches, using different technologies and data rates. Taken together, these studies have suggested that tele-echocardiography assists in the diagnosis and clinical management of patients, avoiding unnecessary transport and potentially reducing costs.^9-12^

More recently, the feasibility of tele-echocardiography for mass screening of heart disease in large communities has been investigated. The exam supposedly detected significant cardiac abnormalities in 16%^3^ to 35%^7^ of the individuals, despite the clear limitation of the different criteria adopted to define heart disease. On the other hand, previous data indicate that focused echocardiography screening tends to overestimate the rate of heart disease in the community, which makes it imperative to validate the examination by an experienced echocardiographist to ensure an adequate level of accuracy.^13^ Even employing well-trained sonographers for local echocardiographic evaluation, remote examination by experienced echocardiographers alters the diagnosis in approximately one quarter of the studies, half of which undergo major clinical changes in the final report.^13^ In general, accuracy appears to be acceptable in the detection of valvular heart disease, although only modest for the diagnosis of systolic dysfunction and left ventricular hypertrophy.^3,5^

In addition to the expansion of cardiovascular imaging through telemedicine, some researchers have also described imaging acquisition through tele-robotics. A French study evaluated 41 individuals with valvular heart disease who underwent tele-echocardiography through a robotic arm operated by an echocardiographer via an internet connection in a room 10 meters away from the patient.^8^ The quality of the images was lower than those obtained by conventional echocardiography, but the diagnosis was reliable in 86% of the cases.^8^ An American study showed the feasibility of carotid vascular ultrasound imaging through the robotic arm and its long-distance transmission over the traditional bandwidth internet.^14^ A Swedish prospective randomized study conducted in a rural community concluded that the combination of cardiologic teleconsultation and robotic arm tele-echocardiography resulted in shorter time to care and diagnosis definition compared to the usual routine referral to the nearest specialty hospital.^15^ However, the number of patients evaluated was small (19 in each group), not allowing inferences regarding clinical outcomes.

Echocardiography as a cardiovascular imaging modality directly depends on the appropriate acquisition and interpretation of satisfactory images. There are no studies conclusively comparing image quality by tele-echocardiography and traditional echocardiography. In parallel, there is no scientific evidence to conclude that the use of tele-echocardiography in primary health care in remote locations is able to reduce morbidity and mortality in the community compared to traditional care workflow.

Obviously, the advent of digital health, which encompasses the use of telemedicine as a useful complementary tool to allow equity of access to health for all Brazilians, is a desirable novelty in the current scenario.^16^ Considering the continental dimension of Brazil, we could assume that populations living in remote areas would benefit from state investment in the spread of digital health. We must welcome the changes that digital transformation can trigger in the practice of medicine, especially where the integrality of access to health is not contemplated. However, such changes should be supported by consistent scientific evidence that accredits them as real advancement, avoiding inappropriate use of new technologies.^17^

There are potential advantages of adopting tele-echocardiography in public healthcare of underprivileged populations in distant locations, but the method still lacks robust scientific validation with prospective controlled studies confirming the health benefits of patients. In addition, a broad discussion on the need for investment in digital technology infrastructure, cost-effectiveness, budgetary impact, regulation and legal certainty, among other challenges and risks, is crucial. It is important to remember that Brazilian law: (a) authorizes only physicians to perform and interpret echocardiograms in the country, and (b) recognizes echocardiography as an area of ​​activity of cardiology and pediatrics. Regulatory debate involving authorities, professional councils and medical societies is mandatory before tele-echocardiography is incorporated into public health policies in Brazil. In the area of ​​supplementary health, there is no legal backing for non-medical individuals, even under the supervision of physicians, to perform echocardiograms, and the use of tele-echocardiography by other health professionals would be a practice not covered by law.^18^ In addition, there is currently no provision for reimbursement for any of the procedures used in Telemedicine, which are not part of the Procedures and Health Events Roll of the National Health Agency. Table 1 lists the potential advantages and challenges for the implementation of tele-echocardiography in Brazil.

**Table 1:** Potential advantages and main challenges for the adoption of tele-echocardiography in Brazil

Potential advantages	Main challenges
Allow access to the method at remote locations	Lack of standardization of tele-echocardiography components and proper internet coverage
Early diagnosis and therapy guidance	Uncertainty whether image quality and diagnostic accuracy is comparable to traditional method
Potential optimization of clinical outcomes	Lack of scientific evidence proving impact on clinical outcomes
Reducing the cost of transporting human resources to geographically distant areas	Absence of scientific evidence showing cost-effectiveness; questions about budget impact and system reimbursement
Reducing the cost of transporting patients to tertiary centers	Uncertainty about adherence by local health professionals
Reduction in the number of unnecessary echocardiograms	Prohibition of Brazilian law to the performance of echocardiography by non-medical operators (sonographers)
Prioritization and organization of waiting lists in healthcare systems with limited availability of specialized exams and consultations	Lack of guidelines for operator training
	Forensic insecurity
	No current laws regarding licensing, data storage, privacy and confidentiality
